# Supplementary material for: Detection of Anaplasma and Ehrlichia bacteria in humans, wildlife, and ticks in the Amazon rainforest
Source: Nat Commun. 2024 May 11;15:3988. doi: 10.1038/s41467-024-48459-y (PMC11088697; doi:10.1038/s41467-024-48459-y)
Supplement: Supplementary file 1 — Supplementary Information [file 41467_2024_48459_MOESM1_ESM.pdf]

SUPPLEMENTARY INFORMATION

Detection of *Anaplasma* and *Ehrlichia* bacteria in humans, wildlife, and ticks in the Amazon rainforest

Buysse *et al*.

Supplementary Table 1. Genovariants of *Ehrlichia*, *Anaplasma*, and *Ca.* Allocryptoplasma detected in French Guiana and best nucleotide identities with 16S rRNA sequences available in GenBank (Last update: 02/14/2024) and obtained this study. The genovariants of *Ehrlichia*, *Anaplasma*, and *Ca.* Allocryptoplasma found in this study were each identified based on their 16S rRNA sequences (1,147-1,150 bp, depending on genovariant).

**Genovariant**	**Host species**	**Best match in public databases (% nucleotide identity; length of the 16S rRNA sequence; query cover; E-value; Genbank accession number)**	**Best match in the present study (% nucleotide identity)**
Ehrlichia #1	*Glyphorynchus spirurus* (wedge-billed woodcreeper)<br>*Pipra aureola* (Crimson-hooded manakin)<br>*Ceratopipra erythrocephala* (golden-headed manakin)<br>*Chiroxiphia pareola* (blue-backed manakin)<br>*Myrmotherula axillaris* (white-flanked antwren)<br>*Amblyomma longirostre* (tick)	*Ehrlichia* sp. clone Ac124 of *Amblyomma calcaratum*, Argentina (99.18%; 1,225 bp; 95%; E=0.0; OQ682485)	*Ehrlichia* genovariant #10 of *Rhipicephalus microplus* (97.43%)
Ehrlichia #2	*Didelphis marsupialis* (common opossum)	*Ehrlichia* sp.MieHl1 of *Haemaphysalis longicornis*, Japan (98.78%; 1,397 bp; 99%; E=0.0; MT258392)	*Ehrlichia* genovariant #3 of *Amblyomma cajennense* (97.74%)
Ehrlichia #3	*Amblyomma cajennense* (Cayenne tick)	*Ehrlichia ruminantium* strain Blaauwkrans (99.13%; 1,484,125 bp; 100%; E=0.0; CP063043)	*Ehrlichia* genovariant #4 of *Amblyomma cajennense* (99.39%)
Ehrlichia #4	*Amblyomma cajennense* (Cayenne tick)	*Ehrlichia* sp. TC251-2 of *Dermacentor nuttalli*, China (99.39%; 1,388 bp; 100%; E=0.0; KJ410253)	*Ehrlichia* genovariant #3 of *Amblyomma cajennense* (99.39%)
Ehrlichia #5	*Hydrochoerus hydrochaeris* (capybara)<br>*Amblyomma romitii* (tick)	*Ehrlichia* sp. HF (98.52%; 1,148,904 bp ; 98.52% ; E=0.0 ; CP007474)	*Ehrlichia* genovariant #3 of *Amblyomma cajennense* (98.78%)
Ehrlichia #6	*Dasypus novemcinctus* (nine-banded armadillo)<br>*Eira barbara* (tayra)	*Ehrlichia* sp. clone irara of *Eira barbara*, Brazil (99.74%; 1,252 bp; 100%; E=0.0; MZ130191)	*Ehrlichia* genovariant #10 of *Rhipicephalus microplus* (98.52%)
Ehrlichia #7	*Amblyomma oblongoguttatum* (tick)	*Ehrlichia ewingii* (99.56%; 1,437 bp ; 100% ; E=0.0 ; U96436)	*Ehrlichia* genovariant #8 of *Amblyomma cajennense* (99.30%)
Ehrlichia #8 (*Ca.* Ehrlichia cajennense) *	*Amblyomma cajennense* (Cayenne tick)	*Ehrlichia* sp. TC251-2 of *Dermacentor nuttalli*, China (99.30%; 1,388 bp; 100%; E=0.0; KJ410253)	*Ehrlichia* genovariant #7 of *Amblyomma oblongoguttatum* (98.52%)
Ehrlichia #9	*Rhipicephalus microplus* (tropical cattle tick)	*Ehrlichia* sp. VKAA024 of *Rhipicephalus microplus*, Malaysia (99.91%; 1,276 bp; 100%; E=0.0; KY046297)	*Ehrlichia* genovariant #10 of *Rhipicephalus microplus* (99.65%)
Ehrlichia #10	*Rhipicephalus microplus* (tropical cattle tick)	*Ehrlichia* sp. clone UN2-100 of *Rhipicephalus microplus*, Malaysia (100%; 1,276bp; 100%; E=0.0; KY046298)	*Ehrlichia* genovariant #9 of *Rhipicephalus microplus* (99.65%)
Anaplasma #11	*Dasypus novemcinctus* (nine-banded armadillo)<br>*Amblyomma cajennense* (Cayenne tick)<br>*Amblyomma oblongoguttatum* (tick)	*Anaplasma platys* strain WHBMXZ-126 (99.74%; 1,282bp; 100%; E=0.0; KX987336)	*Anaplasma* genovariant #13 of human (*Ca.* Anaplasma sparouinense) (97.47%)
Anaplasma #12 (*Anaplasma marginale*)	*Rhipicephalus microplus* (tropical cattle tick)	*Anaplasma marginale* isolate AmCU01 (100%; 1,520bp; 100%; E=0.0; KT264188)	*Anaplasm a*genovariant #14 of *Amblyomma coelebs* (98.08%)
Anaplasma #13 (*Ca.* Anaplasma sparouinense) *	*Homo sapiens* (human)	*Anaplasma marginale* strain WHBMXZ-90-2 (97.91%; 1,281bp; 100%; E=0.0; KX987327)	*Anaplasma* genovariant #14 of *Amblyomma coelebs* (99.99%)
Anaplasma #14	*Amblyomma coelebs* (tick)	*Anaplasma marginale* strain WHBMXZ-90-2 (98.09%; 1,281bp; 100%; E=0.0; KX987327)	*Anaplasma* genovariant #13 of human (*Ca.* Anaplasma sparouinense) (99.99%)
Anaplasma #15 (*Ca.* Anaplasma amazonensis) *	*Bradypus tridactylus* (three-toed sloth)<br>*Choloepus didactylus* (two-toed sloth)	*Anaplasma ovis* isolate Dongwangmang-goat-15 (97.74%; 1,422bp; 100%; E=0.0; MG869525)	*Anaplasm a*genovariant #13 of human (*Ca.* Anaplasma sparouinense) (99.22%)
Ca. Allocryptoplasma #16	*Amblyomma coelebs* (tick)	*Ca.* Allocryptoplasma sp. of the tick *Haemaphysalis parmata* (99.39%; 1,146 bp; 99%; E=0.0; OQ092428)	*Anaplasma* genovariant #11 of armadillo and *Amblyomma* spp. (95.4%)

* Genovariants for which genomes have been sequenced in the present study

Supplementary Table 2. Summary of the assembly information and quality analyses of the newly assembled genomes of *Ca.* Anaplasma sparouinense, *Ca.* Anaplasma amazonensis, and *Ca.* Ehrlichia cajennense. N50 is a measure of contiguity and represents the length at which half of the assembled bases are contained in contigs of equal or greater length. L50 is the number of contigs required to reach or exceed the N50 value (L50 represents the minimum number of contigs needed to cover half of the genome).

Genome	Assembling information									
	Genome size (bp)	Contigs number	N50	L50	Completeness (%)	GC rate (%)	Genes	Pseudogenes	Coding density (%)	Coverage values
Ca. Anaplasma sparouinense (strain Sparouine)	1,187,243	117	20,180	19	97.18	49.43	998	41	96.05	10.57
Ca. Anaplasma amazonensis (strain Petit Saut)	1,176,232	81	30,968	11	97.18	50.46	1,008	43	95.90	11.43
Ca. Ehrlichia cajennense (strain Matoury)	1,177,323	1	1,177,323	1	90.48	32.08	1,402	39	97.30	35.38

Supplementary Table 3. Average Nucleotide Identity (ANI) for *Anaplasma* species and candidatus species. White text on black background indicates ANIs of *Ca.* Anaplasma sparouinense and *Ca.* Anaplasma amazonensis with other *Anaplasma* species.

Genomes	*Ca.* Anaplasma sparouinense (strain Sparouine)	*Ca.* Anaplasma amazonensis (strain Petit Saut)	*Anaplasma capra* (strain BIME1) (JAOTBF010000000)	*Anaplasma centrale* (strain Israel) (CP001759)	*Anaplasma marginale* (strain St Maries) (CP000030)	*Anaplasma marginale* (strain Florida) (CP001079)	*Anaplasma ovis* (strain Haibei) (CP015994)	*Anaplasma phagocytophilum* (strain Norway variant2) (CP015376)	*Anaplasma phagocytophilum* (strain Norway variant1) (CP046639)	*Anaplasma phagocytophilum* (strain HZ) (CP000235)	*Anaplasma phagocytophilum* (strain JM) (CP006617)	*Anaplasma platys* (strain S3) (CP046391)
Ca. Anaplasma sparouinense (strain Sparouine)	100,00%											
Ca. Anaplasma amazonensis (strain Petit Saut)	88,26%	100,00%										
Anaplasma capra (strain BIME1) (JAOTBF010000000)	85,44%	87,44%	100,00%									
Anaplasma centrale (strain Israel) (CP001759)	84,15%	84,68%	83,08%	100,00%								
Anaplasma marginale (strain St Maries) (CP000030)	85,58%	84,99%	82,74%	91,29%	100,00%							
Anaplasma marginale (strain Florida) (CP001079)	85,57%	85,17%	82,76%	91,30%	99,14%	100,00%						
Anaplasma ovis (strain Haibei) (CP015994)	85,72%	85,44%	82,99%	85,36%	87,28%	87,33%	100,00%					
Anaplasma phagocytophilum (strain Norway variant2) (CP015376)	89,98%	89,83%	91,94%	85,66%	87,84%	87,44%	87,25%	100,00%				
Anaplasma phagocytophilum (strain Norway variant1) (CP046639)	88,34%	89,79%	91,88%	87,97%	87,86%	87,45%	87,53%	96,63%	100,00%			
Anaplasma phagocytophilum (strain HZ) (CP000235)	88,59%	90,21%	91,65%	85,64%	86,73%	86,39%	86,37%	96,74%	95,83%	100,00%		
Anaplasma phagocytophilum (strain JM) (CP006617)	88,60%	90,24%	91,66%	85,63%	86,73%	86,39%	86,40%	96,72%	95,83%	99,73%	100,00%	
Anaplasma platys (strain S3) (CP046391)	88,17%	88,91%	91,48%	91,46%	92,05%	91,75%	91,63%	88,93%	87,98%	88,40%	88,79%	100,00%

Supplementary Table 4. Average Nucleotide Identity (ANI) for *Ehrlichia* species and candidatus species. White text on black background indicates ANIs of *Ca.* Ehrlichia cajennense with other *Ehrlichia* species.

Genomes	*Ca.* Ehrlichia cajennense (strain Matoury)	*Ehrlichia canis* (strain YZ-1) (CP025749)	*Ehrlichia canis* (strain Jake) (CP000107)	*Ehrlichia minasensis* (strain B11) (QOHL01000000)	*Ehrlichia chaffeensis* (strain Liberty) (CP007476)	*Ehrlichia chaffeensis* (strain Arkansas) (CP000236)	*Ehrlichia chaffeensis* (strain West Paces) (CP007480)	*Ehrlichia* sp. HF (CP007474)	*Ehrlichia muris* (strain AS145) (CP006917)	*Ehrlichia ruminantium* (strain Springbokfontein5) (CP040113)	*Ehrlichia ruminantium* (strain Nonile) (CP040117)	*Ehrlichia ruminantium* (strain Welgevonden) (CR767821)	*Ehrlichia ruminantium* (strain Springbokfontein1) (CP040116)
Ca. Ehrlichia cajennense (strain Matoury)	100,00%												
Ehrlichia canis (strain YZ-1) (CP025749)	83,65%	100,00%											
Ehrlichia canis (strain Jake) (CP000107)	83,66%	99,76%	100,00%										
Ehrlichia minasensis (strain B11) (QOHL01000000)	83,71%	90,79%	90,78%	100,00%									
Ehrlichia chaffeensis (strain Liberty) (CP007476)	83,79%	85,76%	85,75%	85,59%	100,00%								
Ehrlichia chaffeensis (strain Arkansas) (CP000236)	83,79%	85,74%	85,75%	85,58%	99,45%	100,00%							
Ehrlichia chaffeensis (strain West Paces) (CP007480)	83,82%	85,77%	85,76%	85,59%	99,45%	99,51%	100,00%						
Ehrlichia sp. HF (CP007474)	83,70%	85,84%	85,84%	85,63%	87,45%	87,43%	87,43%	100,00%					
Ehrlichia muris (strain AS145) (CP006917)	83,84%	85,82%	85,80%	85,77%	87,29%	87,27%	87,25%	92,46%	100,00%				
Ehrlichia ruminantium (strain Springbokfontein5) (CP040113)	83,40%	83,62%	83,63%	83,67%	83,58%	83,59%	83,56%	83,54%	83,45%	100,00%			
Ehrlichia ruminantium (strain Nonile) (CP040117)	83,33%	83,59%	83,59%	83,62%	83,37%	83,39%	83,37%	83,52%	83,53%	97,92%	100,00%		
Ehrlichia ruminantium (strain Welgevonden) (CR767821)	83,28%	83,56%	83,57%	83,60%	83,41%	83,43%	83,41%	83,54%	83,55%	97,90%	99,83%	100,00%	
Ehrlichia ruminantium (strain Springbokfontein1) (CP040116)	83,24%	83,58%	83,58%	83,63%	83,41%	83,43%	83,41%	83,52%	83,49%	97,89%	99,80%	99,89%	100,00%

Supplementary Table 5. Primers used in polymerase chain reaction (PCR) assays and Sanger sequencing for 16S rDNA and *gltA* typing of *Ehrlichia*, *Anaplasma* and *Ca.* Allocryptoplasma infections.

Gene	Product	Primers (5'-3')	Fragment size	Reference
16S rDNA	Small ribosomal subunit (SSU rRNA)	For *Anaplasma* spp.:		
		Ehr16S_F1 - TCGCTATTAGATGAGCCTA	1st round PCR: Ehr16S_F1 / Ehr16S_R3 : 1187bp	[1] ; this study
		Anap16S_Fint - CTGGTCCGGTACTGACGCTGAG	2nd round PCR (fragment 1): Ehr16S_F1 / Anap_16S_Rint : 623bp	
		Ehr16S_R3 - AGTTAAGCCAATTCCCATGG	2nd round PCR (fragment 2): Anap_16S_Fint / Ehrlichia_R3 : 691bp	
		Anap16S_Rint - CGCGTTAGCTACAACACAGAG		
		For *Ehrlichia* spp. and *Ca.* Allocryptoplasma:		[1] ; this study
		Ehr16S_F1 - TCGCTATTAGATGAGCCTA	1st round PCR: Ehr16S_F1 / Ehr16S_R2 : 1202bp	
		Ehr16S_F2 - TGACATGAAGGTCGTATCC	2nd round PCR (fragment 1): Ehr16S_F1 / Ehr16S_R1 : 796bp	
		Ehr16S_R1 - GGTCCAGCCGAACTGACTC	2nd round PCR (fragment 2): Ehr16S_F2 / Ehr16S_R3 : 442bp	
		Ehr16S_R2 - AGCACACCAGCTTCGAGTTA		
		Ehr16S_R3 - AGTTAAGCCAATTCCCATGG		
		For *Ehrlichia* spp.:		
gltA	Citrate synthetase	EgltAf1 - TAACTTATGATCCAGGNTTT	1st round PCR: EgltAf1 / EgltAr : 517-523 bp	This study
		EgltAf2 - ATGTCTACTGCTGCTTGT	2d round PCR: EgltAf2 / EgltAr : 497-503 bp	
		EgltAr - TCATGATCAGCATGYAAT		

Semi-nested PCR amplifications were performed as follows: the first PCR run with the external primers was performed in a 10 µL volume containing 10–50 ng of genomic DNA, 3 mM of each dNTP (Thermo Scientific), 8 mM of MgCl2 (Roche Diagnostics), 3 µM of each primer, 1 µL of 10× PCR buffer (Roche Diagnostics), and 0.5 U of Taq DNA polymerase (Roche Diagnostics). A 1 µL aliquot of the PCR product from the first reaction was then used as a template for the second round of amplification. The second PCR was performed in a total volume of 25 µL and contained 8 mM of each dNTP (Thermo Scientific), 10 mM of MgCl2 (Thermo Scientific), 7.5 µM of each of the internal primers, 2.5 µL of 10×PCR buffer (Thermo Scientific), and 1.25 U of Taq DNA polymerase (Thermo Scientific). All PCR amplifications were performed as follows: initial denaturation at 93 °C for 3 min, 35 cycles of denaturation (93 °C, 30 s), annealing (T m = 52 °C), extension (72 °C, 1 min), and a final extension at 72 °C for 5 min. Positive (DNA of specimens infected either by *Anaplasma* spp. or by *Ehrlichia* spp.) and negative (water) controls were included in each PCR assay.

Reference

1. Lacroux, C. *et al.* Survey of ticks and tick-borne pathogens in wild chimpanzee habitat in Western Uganda. *Parasit. Vectors* **16,** 22 (2023).

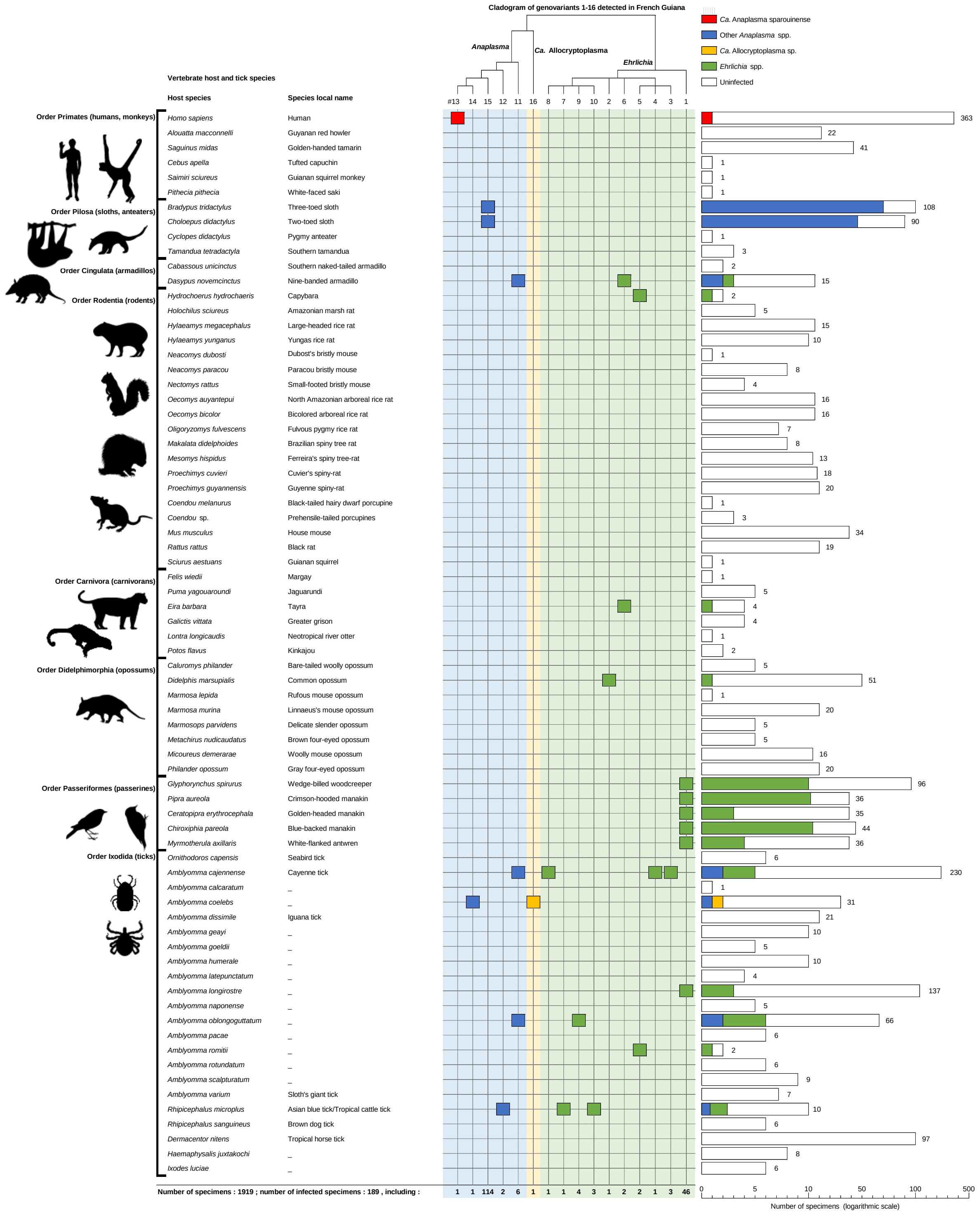

Supplementary Figure 1. Distribution of *Ehrlichia*, *Anaplasma*, and *Ca.* Allocryptoplasma infections in humans, wildlife, and associated ticks in French Guiana. The left part of the Figure shows the list of 72 species under investigation (see Figure 2 for visualization of only the 18 infected species). The top part shows a cladogram of the *Ehrlichia*, *Anaplasma*, and *Ca.* Allocryptoplasma genovariants detected in this study. The right part of the figure gives the distribution of infections in specimens for each of the 72 examined species (color: infected specimens; white: uninfected specimens).

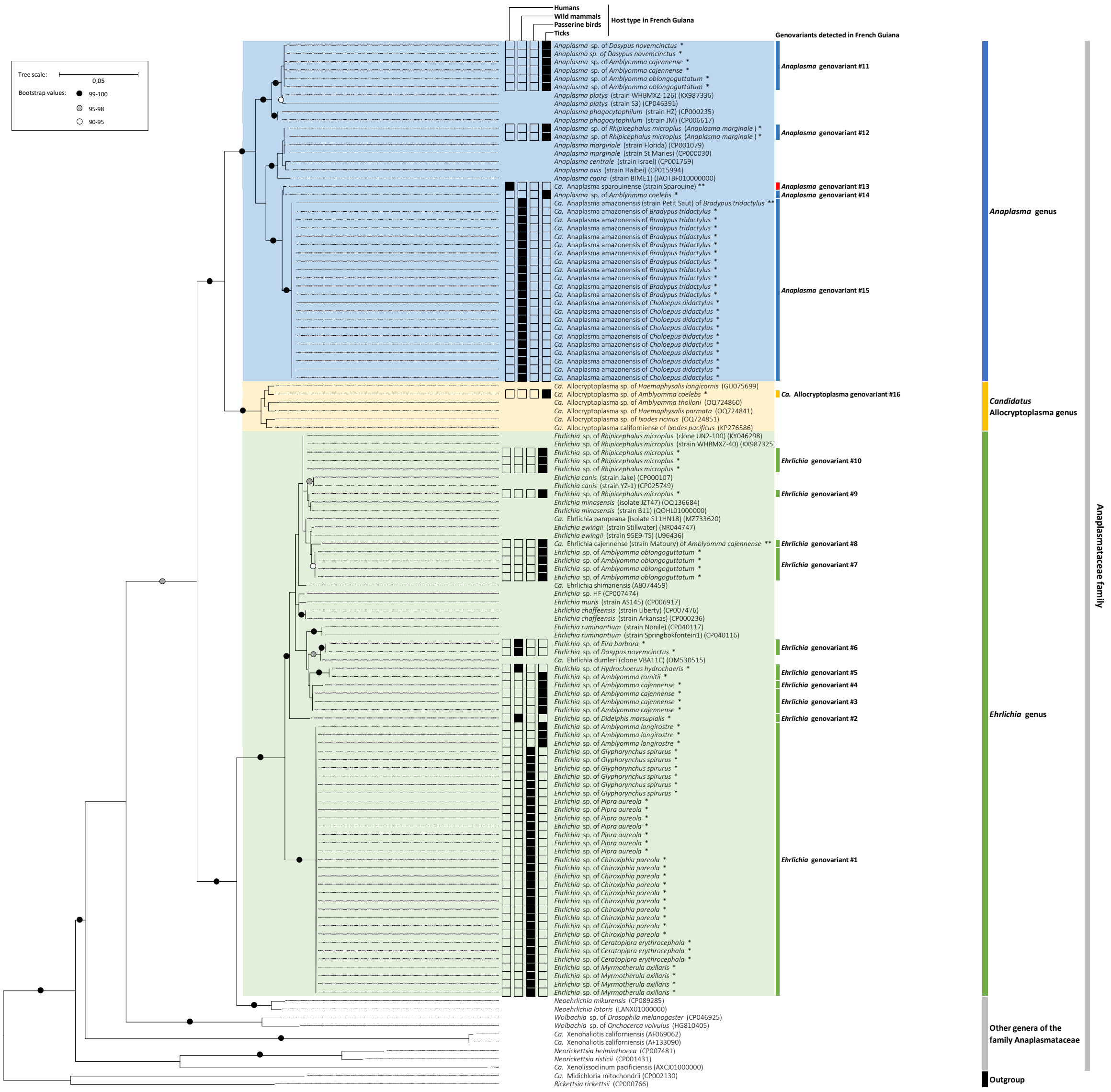

Supplementary Figure 2. Phylogeny of the family Anaplasmataceae constructed using maximum-likelihood (ML) estimations based on 16S rDNA sequences with a total of 1,149 unambiguously aligned bp (best-fit approximation for the evolutionary model: GTR+G+I). All sequences produced in this study have been including in the phylogenetic tree (see Figure 3 for visualization of only one sequence per genovariant and per host species). For *Ehrlichia*, *Anaplasma*, and *Ca.* Allocryptoplasma detected in French Guiana, host type (humans, wild mammals, passerines or ticks) is shown by black squares. *, 16S rDNA sequences of *Ehrlichia*, *Anaplasma*, and *Ca.* Allocryptoplasma obtained in this study; **, *Ca.* Anaplasma sparouinense, *Ca.* Anaplasma amazonensis, and *Ca.* Ehrlichia cajennense MAGs obtained in this study. GenBank accession numbers of other sequences used in analyses are shown on the phylogenetic trees. Numbers at nodes indicate bootstrap support percentage with 1,000 replicates. Only bootstrap values >90% are shown. The scale bar is in units of mean number of substitutions/site.

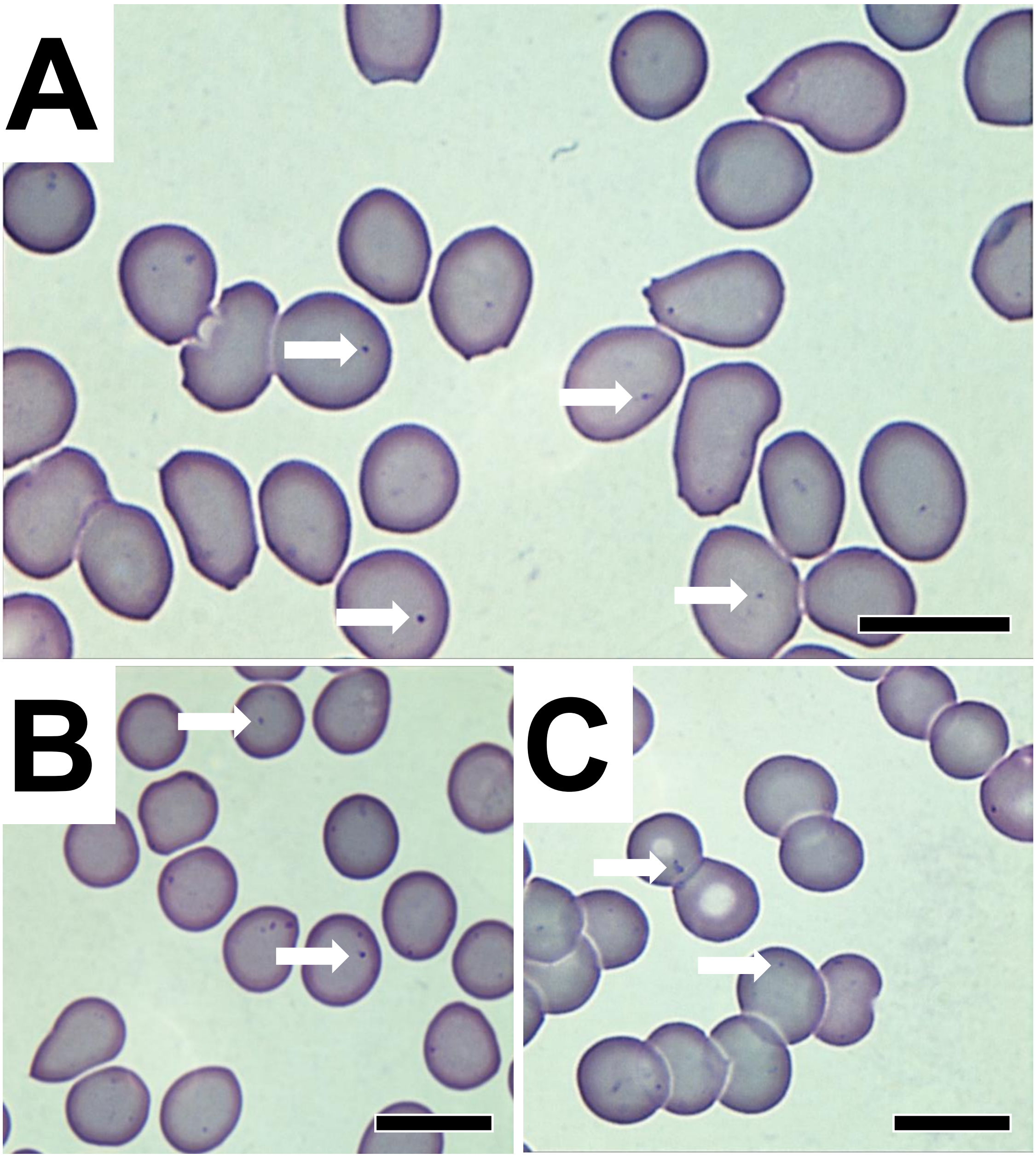

Supplementary Figure 3. Thin blood films from a patient infected by *Ca.* Anaplasma sparouinense. **A, B, C** The bacteria are located at the periphery of the red blood cells as small round dots of 0.3–0.4 µm (white arrow). Wright-Giemsa stain; original magnification ×100; black bar: 5µm.

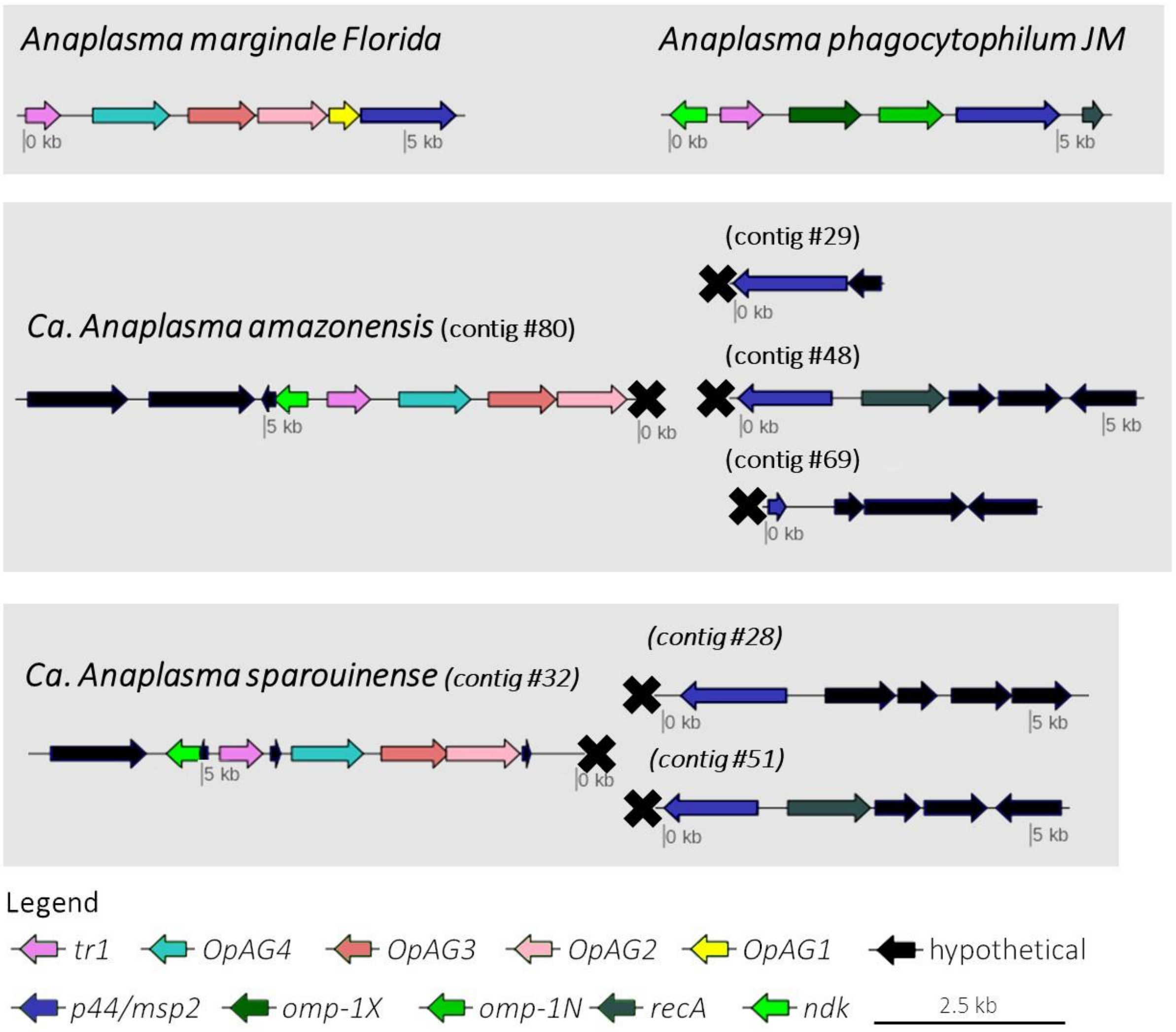

Supplementary Figure 4. The *msp2*/*p44* loci. The *Ca.* Anaplasma sparouinense and *Ca.* Anaplasma amazonensis genomes each contain single copies of the transcriptional regulator 1 (*tr1*) and three operon-associated (*OpAG4* [=*OMP1*], *OpAG3* and *OpAG2*) genes, typically situated downstream of the *msp2*/*p44* expression locus in *Anaplasma marginale* genomes. The *ndk* gene is present upstream of *tr1* in the genome of *Anaplasma marginale* Florida strain and is not shown. *OpAG3* and *OpAG2* are part of the *msp2* operon, whilst *OpAG4* is most likely transcribed independently. In each *Anaplasma* genome sequenced in this study, we identified only one contig with the *tr1*, *OpAG4*, *OpAG3* and *OpAG2* (at the end of the contig), alongside several contigs containing *msp2*/*p44* copies (where *msp2*/*p44* sequences often are at the end of contigs). The figures solely depict *msp2*/*p44* sequences located at the termini of contigs. Black crosses denote the contig ends.
