# Supplementary material for: Detection of Anaplasma and Ehrlichia bacteria in humans, wildlife, and ticks in the Amazon rainforest
Source: Nat Commun. 2024 May 11;15:3988. doi: 10.1038/s41467-024-48459-y (PMC11088697; doi:10.1038/s41467-024-48459-y)
Supplement: Supplementary file 3 — Reporting Summary [file 41467_2024_48459_MOESM3_ESM.pdf]

nature portfolio

Corresponding author(s): Olivier DURON

Last updated by author(s): Apr 5, 2024

Reporting Summary

Nature Portfolio wishes to improve the reproducibility of the work that we publish. This form provides structure for consistency and transparency in reporting. For further information on Nature Portfolio policies, see our Editorial Policies and the Editorial Policy Checklist.

Statistics

For all statistical analyses, confirm that the following items are present in the figure legend, table legend, main text, or Methods section.

n/a	Confirmed	
☐	☒	The exact sample size (*n*) for each experimental group/condition, given as a discrete number and unit of measurement
☐	☒	A statement on whether measurements were taken from distinct samples or whether the same sample was measured repeatedly
☐	☒	The statistical test(s) used AND whether they are one- or two-sided<br>*Only common tests should be described solely by name; describe more complex techniques in the Methods section.*
☐	☒	A description of all covariates tested
☐	☒	A description of any assumptions or corrections, such as tests of normality and adjustment for multiple comparisons
☐	☒	A full description of the statistical parameters including central tendency (e.g. means) or other basic estimates (e.g. regression coefficient) AND variation (e.g. standard deviation) or associated estimates of uncertainty (e.g. confidence intervals)
☐	☒	For null hypothesis testing, the test statistic (e.g. *F*, *t*, *r*) with confidence intervals, effect sizes, degrees of freedom and *P* value noted<br>*Give P values as exact values whenever suitable.*
☒	☐	For Bayesian analysis, information on the choice of priors and Markov chain Monte Carlo settings
☒	☐	For hierarchical and complex designs, identification of the appropriate level for tests and full reporting of outcomes
☒	☐	Estimates of effect sizes (e.g. Cohen's *d*, Pearson's *r*), indicating how they were calculated

Our web collection on statistics for biologists contains articles on many of the points above.

Software and code

Policy information about availability of computer code

Data collection	No software was used for data collection.
Data analysis	All command lines and sotwares used in our analyses are detailled in a README file deposited in a repository on GitHub (see https://github.com/mariebuysse/Anaplasmataceae_ms). The DOI for our Github repository is: https://zenodo.org/doi/10.5281/zenodo.10911528. All the softwares used in the present study are also listed in the 'Methods' section in the current version of the ms. The bibliographical references describing them and the versions used for analysis are also provided. No software was developed for our analyses. Here is the list of data collection/data analysis software/tools/algorithms/packages used in the study: Prokka (v1.13.1) CGView (v1.5), Pseudofinder (v1.0), OrthoFinder (v2.3.12), MAFFT (v7.450), trimAl (v1.2rev59), AMAS (v1.01), ClustalW (1.2.2), DNASP (6.12.03), FastQC (0.12.0), Atropos (1.1), Albacore v2.0.1, MEGAHIT (v1.2.9), Concoct (v1.1.0), Flye (v2.4.1), Medaka tool (v1.5.0), Bandage (v0.8.1), QUAST (v4.6.3), miComplete (v1.1.1), modeltest-ng (v0.1.5), RAxML (v8.2.9). Further statistical analyses were carried out using R (https://www.r-project.org/).

For manuscripts utilizing custom algorithms or software that are central to the research but not yet described in published literature, software must be made available to editors and reviewers. We strongly encourage code deposition in a community repository (e.g. GitHub). See the Nature Portfolio guidelines for submitting code & software for further information.

Data

Policy information about availability of data

All manuscripts must include a data availability statement. This statement should provide the following information, where applicable:

- Accession codes, unique identifiers, or web links for publicly available datasets
- A description of any restrictions on data availability
- For clinical datasets or third party data, please ensure that the statement adheres to our policy

The 16S rDNA and gltA nucleotide sequences generated in this study have been deposited in the GenBank database under accession code OR854269-OR854350 and PP400975- PP400976 (https://www.ncbi.nlm.nih.gov/genbank/). The raw genomic data and metagenome-Assembled Genomes (MAGs) produced and analyzed in our study are available in Genome Sequence Archive (GSA) database under project PRJCA019774 (https://ngdc.cncb.ac.cn/gsa/). Source data on infection distribution and prevalence generated in this study are provided with this paper in the Source Data File 1.

Each OTU sequence was aligned and taxonomically assigned using the Silva database (https://www.arb-silva.de/) allowing the detection of Anaplasmataceae bacterial reads. Assignation of genomes was next confirmed using the online NCBI BLAST tool (https://blast.ncbi.nlm.nih.gov/Blast.cgi).
Ankyrin (ANK) domains and porin proteins from the OMP1 superfamily were identified using the NCBI Conserved Domains Database (CDD, https://www.ncbi.nlm.nih.gov/Structure/cdd) and the SMART database (http://smart.embl-heidelberg.de/).

Research involving human participants, their data, or biological material

Policy information about studies with human participants or human data. See also policy information about sex, gender (identity/presentation), and sexual orientation and race, ethnicity and racism.

Reporting on sex and gender	No sex or gender data has been collected for this study.
Reporting on race, ethnicity, or other socially relevant groupings	No data on race or ethnicity were collected for this study. However, part of this study focuses on humans living in the depths of the French Guyanese rainforests, and more specifically on gold miners, as explained in the ms. We examined this population for the presence of pathogens circulating in rainforests.
Population characteristics	The other population characteristics of the human research participants as age have been recorded and reported in a previous study (https://doi.org/10.1186/s12889-017-4557-4), but these data have not been used in the present study and have not been presented/considered/examined/analyszed in this context.
Recruitment	Because of the remoteness of mining camps, the blood sampling was implemented at 'resting sites', which are transborder areas located in Surinam, on its eastern border with French Guiana. Gold miners go to these informal settlements for transactions of gold and logistical supplies. A previous study along the borders of French Guiana demonstrated that these sites are strategic to efficiently target this highly mobile population for public health actions.
Ethics oversight	Human samples were collected in Suriname with the approval of the National Ethics Board of Suriname (CMWO (Commissie voor Mensgebonden Wetenschappelijk Onderzoek), Opinion Number VG 25-17). The authorization of importation of human biological samples to France was obtained and the biological collection declared to the French Ministry of Education and Research (DC-2021-4649). The database was anonymized and registered to the Data Protection Officer according to the General Data Protection Regulation (GDPR).

Note that full information on the approval of the study protocol must also be provided in the manuscript.

Field-specific reporting

Please select the one below that is the best fit for your research. If you are not sure, read the appropriate sections before making your selection.

☒ Life sciences ☐ Behavioural & social sciences ☐ Ecological, evolutionary & environmental sciences

For a reference copy of the document with all sections, see nature.com/documents/nr-reporting-summary-flat.pdf

Life sciences study design

All studies must disclose on these points even when the disclosure is negative.

Sample size	No sample size calculation was made. We have used collections of already published samples, previously used for other purposes. Since no sampling was done for this study, our data was thus constrained by what was already available in collections. We listed the previous papers describing these collections in the 'Methods' section of the ms. Our survey is not an experimental study with experimental units (such as participants, subjects, or samples) assigned to different treatment groups or conditions.
Data exclusions	No data was excluded from the analyses.
Replication	Our study does not include any parts subject to replication analyses. Our data are mostly based on genetic and genomic typing of tick-borne bacteria. Our survey is not an experimental study with experimental units (such as participants, subjects, or samples) assigned to different treatment groups or conditions, meaning that replication is not relevant here.

Randomization	Our survey is not an experimental study with experimental units (such as participants, subjects, or samples) assigned to different treatment groups or conditions, meaning that randomization is not relevant here.
Blinding	Our survey is not an experimental study with experimental units (such as participants, subjects, or samples) assigned to different treatment groups or conditions, meaning that blinding is not relevant here.

Reporting for specific materials, systems and methods

We require information from authors about some types of materials, experimental systems and methods used in many studies. Here, indicate whether each material, system or method listed is relevant to your study. If you are not sure if a list item applies to your research, read the appropriate section before selecting a response.

Materials & experimental systems

n/a	Involved in the study
☒	☐ Antibodies
☒	☐ Eukaryotic cell lines
☒	☐ Palaeontology and archaeology
☐	☒ Animals and other organisms
☒	☐ Clinical data
☒	☐ Dual use research of concern
☒	☐ Plants

Methods

n/a	Involved in the study
☒	☐ ChIP-seq
☒	☐ Flow cytometry
☒	☐ MRI-based neuroimaging

Animals and other research organisms

Policy information about studies involving animals; ARRIVE guidelines recommended for reporting animal research, and Sex and Gender in Research

Laboratory animals	n/a
Wild animals	No animals were captured for this study. All samples were taken from already established and published collections for reasons other than those covered by the present study. Birds were netted in the forest and released immediately after collection of measurements, blood samples and ticks. Some non-human mammals were captured in the forest (notably by trapping) and released immediately after sampling into the wild. Other non-human mammals were found dead (e.g. recently road-killed animals) and used as such for sampling. All sampled species are listed in the MS, as further detailed in Table S1 (with sample size, sampling locations, types of biological material used, infection status, etc.).
Reporting on sex	n/a in this study
Field-collected samples	No samples collected in the field were brought to the laboratory alive. All blood samples were taken in the field. Biopsies were taken either in the field or in the laboratory (on dead animals collected in the field).
Ethics oversight	The organizations that authorized this protocol and sampling of wildlife included French government structures and NGO. They are all listed in the ms. These organizations are: French Ministry of Education and Research, French Ministry of the Environment, Prefecture of Guyane, Convention on International Trade in Endangered Species of Wild Fauna and Flora (CITES), Pasteur Institute, the Direction de l'Environnement, de l'Aménagement et du Logement (DEAL) de Guyane, the Direction Régionale de l'Office National des Forêts (ONF) de Guyane, the Conservatoire du Littoral, the Centre National d'Etudes Spatiales (CNES), the Centre Spatial Guyanais (CSG), the Association pour la Découverte de la Nature en Guyane, the association Randoroura.

Note that full information on the approval of the study protocol must also be provided in the manuscript.
